# (*E*)-1-[2-(2-Cyano­phen­yl)diazen-2-ium-1-yl]naphthalen-2-olate

**DOI:** 10.1107/S1600536813017261

**Published:** 2013-06-29

**Authors:** Hassiba Bougueria, Mohamed Amine Benaouida, Sofiane Bouacida, Abd el kader Bouchoul

**Affiliations:** a Unité de Recherche de Chimie de l’Environnement et Moléculaire Structurale (CHEMS), Département de Chimie, Université Mentouri de Constantine 1, 25000 Constantine, Algeria

## Abstract

There are two independent zwitterion mol­ecules (*A* and *B*) in the asymmetric unit of the title compound, C_17_H_11_N_3_O, which belongs to the family of azo dyes. The dihedral angle between the benzene ring and the naphthalene ring system is 6.99 (6)° in mol­ecule *A* and 4.38 (6)° in mol­ecule *B*. The azo group adopts an *E* conformation with respect to the –N=N– bond and each of the independent mol­ecules has an intra­molecular N—H⋯O hydrogen bond. In the crystal, mol­ecules are linked by C—H⋯O and C—H⋯N hydrogen bonds, forming ribbons propagating along [-110]. The ribbons are linked *via* π–π inter­actions involving the benzene and naphthalene rings of inversion-related *A* and inversion-related *B* mol­ecules, forming a three-dimensional structure. The most significant centroid–centroid distances vary from 3.6599 (6) to 3.7538 (9) Å.

## Related literature

For general background to azo compounds and their use in dyes, pigments and advanced materials, see: Lee *et al.* (2004 ▶); Oueslati *et al.* (2004 ▶). Many azo compounds have been synthesized by diazo­tization and diazo coupling reactions, see: Wang *et al.* (2003 ▶). For a related structure, see: Rãdulescu *et al.* (2006 ▶). For bond-length data, see: Allen *et al.* (1987 ▶).
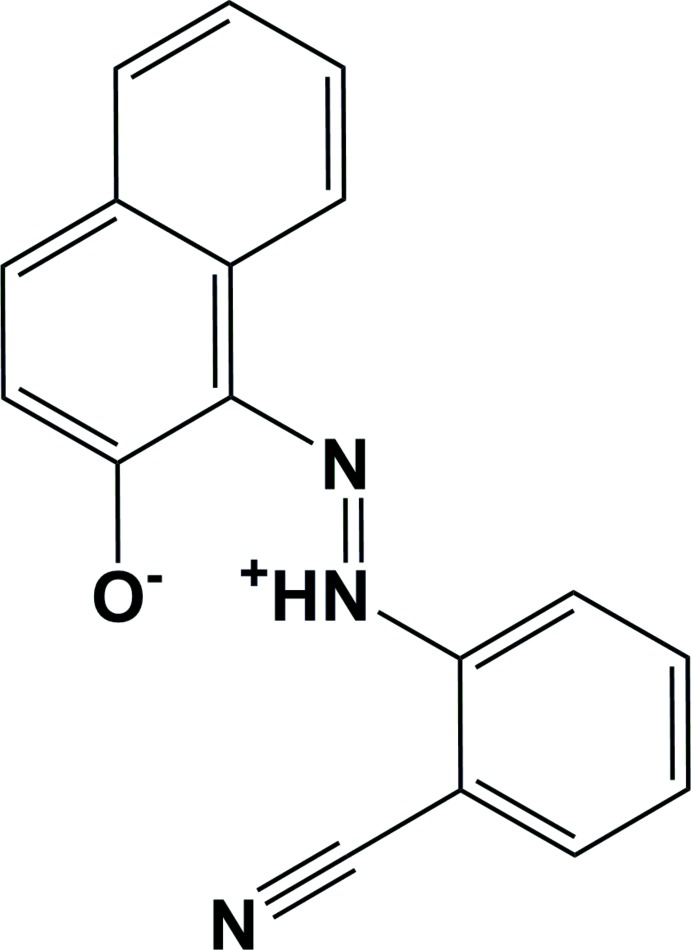


## Experimental

### 

#### Crystal data

C_17_H_11_N_3_O
*M* *_r_* = 273.29
Triclinic, 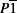

*a* = 7.1296 (3) Å
*b* = 12.9532 (7) Å
*c* = 15.6181 (8) Å
α = 111.562 (2)°
β = 90.536 (2)°
γ = 100.779 (2)°
*V* = 1312.92 (11) Å^3^
*Z* = 4
Mo *K*α radiation
μ = 0.09 mm^−1^
*T* = 150 K
0.55 × 0.11 × 0.08 mm

#### Data collection

Bruker APEXII diffractometer
Absorption correction: multi-scan (*SADABS*; Sheldrick, 2002 ▶) *T*_min_ = 0.910, *T*_max_ = 0.993
18729 measured reflections
6021 independent reflections
3859 reflections with *I* > 2σ(*I*)
*R*_int_ = 0.037

#### Refinement

*R*[*F* ^2^ > 2σ(*F* ^2^)] = 0.046
*wR*(*F* ^2^) = 0.120
*S* = 1.06
6021 reflections
387 parameters
2 restraints
H atoms treated by a mixture of independent and constrained refinement
Δρ_max_ = 0.20 e Å^−3^
Δρ_min_ = −0.23 e Å^−3^

### 

Data collection: *APEX2* (Bruker, 2006 ▶); cell refinement: *SAINT* (Bruker, 2006 ▶); data reduction: *SAINT*; program(s) used to solve structure: *SIR97* (Altomare *et al.*, 1999 ▶); program(s) used to refine structure: *SHELXL97* (Sheldrick, 2008 ▶); molecular graphics: *ORTEP-3 for Windows* (Farrugia, 2012 ▶); software used to prepare material for publication: *WinGX* (Farrugia, 2012 ▶).

## Supplementary Material

Crystal structure: contains datablock(s) global, I. DOI: 10.1107/S1600536813017261/su2616sup1.cif

Structure factors: contains datablock(s) I. DOI: 10.1107/S1600536813017261/su2616Isup2.hkl

Supplementary material file. DOI: 10.1107/S1600536813017261/su2616Isup3.cml

Additional supplementary materials:  crystallographic information; 3D view; checkCIF report

## Figures and Tables

**Table 1:** Hydrogen-bond geometry (Å, °)

*D*—H⋯*A*	*D*—H	H⋯*A*	*D*⋯*A*	*D*—H⋯*A*
N2—H2⋯O1	0.92 (1)	1.80 (2)	2.5380 (16)	136 (2)
N5—H5⋯O2	0.90 (1)	1.72 (2)	2.5277 (17)	147 (2)
C21—H21⋯N3^i^	0.93	2.61	3.509 (2)	162
C30—H30⋯N3^ii^	0.93	2.60	3.487 (2)	159
C32—H32⋯O1^iii^	0.93	2.49	3.1994 (18)	133

Symmetry codes: (i) 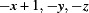; (ii) 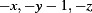; (iii) 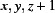.

**Fig. 1.**
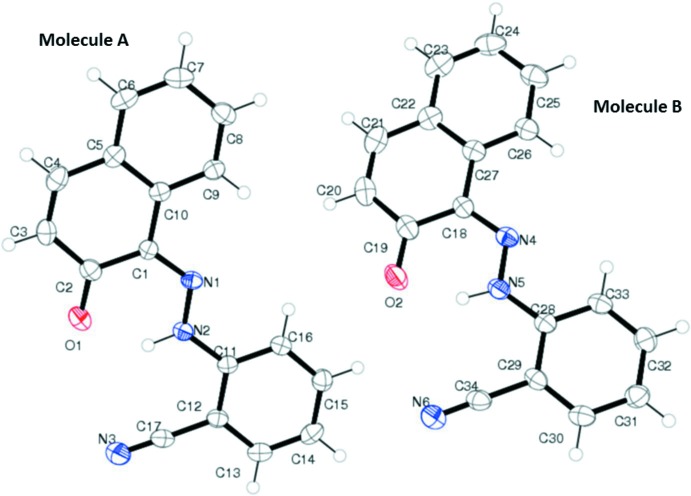
The molecular structure of the two independent molecules (A and B) of the title compound, with atom labelling. Displacement ellipsoids are drawn at the 50% probability level.

**Fig. 2.**
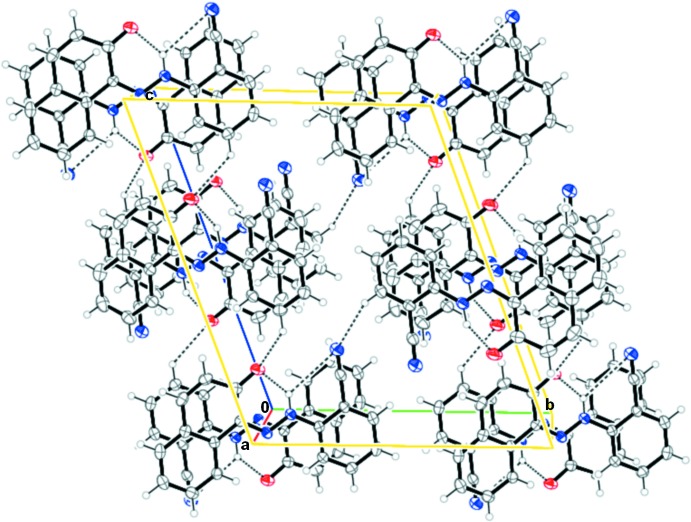
A view along the a axis of the crystal packing of the title compound, showing the hydrogen bonds as dashed lines (see Table 1 for details).

## supplementary crystallographic information

### Comment

Azo dno's (dyes and pigments) are by far the most important class of dno's, accounting for over 50% of all commercial dno's, and having been studied more than any other class of dye. Azo dno's contain at least one azo group (–N=N–) but can contain two (diazo), three (triazo), or more but rarely, four (tetrakisazo) or more (polyazo) azo groups. The azo group is attached to two groups of which at least one, but more usually both, are aromatic. They exist in the *trans* form in which the band angle vis. 120°; the nitrogen atoms are sp^2^ hybridized. Almost without exception, azo dno's are made by diazotization of a primary aromatic amine followed by coupling of the resultant diazonium salt with an electron-rich nucleophile (Wang *et al.*, 2003). We report herein on the crystal structure of the title compound, obtained through the diazotization of 2-cyanoaniline followed by a coupling reaction with 2-naphthol.

The molecular structure of the title compound is shown in Fig. 1. The asymmetric unit contains two independent molecules (A and B) with no significant differences in their structures. The bond distances (Allen *et al.*, 1987) and bond angles in the two molecules are normal and similar to those in a related compound (Rãdulescu *et al.*, 2006). Interestingly, the hydrogen atom of the OH group has been transfered to the N atom in the azo group to form a zwitterion, and in each of the independent molecules there is an intramolecular N—H···O hydrogen bond (Table 1). The molecules are relatively planar with the dihedral angle between the benzene ring and naphthalene ring system being 6.99 (6) ° in A and 4.38 (6) ° in B. Both molecules have an *E* conformation with respect to azo bridge (Fig. 1). The C1-N1-N2-C11 torsion angle is -175.64 (12) ° in A and the C18–N4–N5–C28 torsion angle is-177.81 (13)° in B, confirming the *trans* conformation of the C atom with respect to hydrazine N atom.

In the crystal, molecules are linked by C-H···O and C-H···N hydrogen bonds forming ribbons propagating along [-110]; see Table 1 and Fig. 2. The ribbons are linked via π-π interactions involving the benzene and naphthalene rings of inversion related A and inversion related B molecules. The most significant centroid-to-centroid distances are Cg1···Cg3^i^ and Cg2···Cg3^i^ = 3.6636 (9) and 3.7538 (9) Å, respectively, for the A molecules, and Cg5···Cg7^ii^ and Cg6···Cg7^ii^ = 3.6599 (6) and 3.6610 (9) Å, respectively, for the B molecules [Cg1, Cg2, Cg3, Cg5, Cg6 and Cg7 are the centroids of the C1-C5/C10, C5-C10, C11-C16, C18-C22/C27, C22-C27 and C28-C30 rings, respectively; symmetry codes: (i) -x+1, -y, -z; (ii) -x, -y, -z+1].

### Experimental

The title compound was obtained through the diazotization of 2-cyanoaniline followed by a coupling reaction with 2-naphthol, according to the literature procedure used to synthesize other aromatic azo-compounds (Wang *et al.*, 2003). Orange rod-like crystals of the title compound were obtained by slow evaporation at room temperature of a solution in H_2_O/THF (1/1 *v*/*v*).

### Refinement

The NH H atoms were located in a difference Fourier map and refined with distance restraints [N-H = 0.89 (1) Å]. The C-bound H atoms were included in calculated positions and treated as riding atoms: C-H = 0.93 Å with U_iso_(H) = 1.2U_eq_(C).

### Crystal data

**Table:**

C_17_H_11_N_3_O	*Z* = 4
*M**_r_* = 273.29	*F*(000) = 568
Triclinic, *P*1	*D*_x_ = 1.383 Mg m^−^^3^
Hall symbol: -P 1	Mo *K*α radiation, λ = 0.71073 Å
*a* = 7.1296 (3) Å	Cell parameters from 4069 reflections
*b* = 12.9532 (7) Å	θ = 2.7–27.2°
*c* = 15.6181 (8) Å	µ = 0.09 mm^−^^1^
α = 111.562 (2)°	*T* = 150 K
β = 90.536 (2)°	Rod, orange
γ = 100.779 (2)°	0.55 × 0.11 × 0.08 mm
*V* = 1312.92 (11) Å^3^	

### Data collection

**Table:**

Bruker APEXII diffractometer	6021 independent reflections
Radiation source: fine-focus sealed tube	3859 reflections with *I* > 2σ(*I*)
Graphite monochromator	*R*_int_ = 0.037
CCD rotation images, thin slices scans	θ_max_ = 27.6°, θ_min_ = 1.4°
Absorption correction: multi-scan (*SADABS*; Sheldrick, 2002)	*h* = −9→8
*T*_min_ = 0.910, *T*_max_ = 0.993	*k* = −15→16
18729 measured reflections	*l* = −20→19

### Refinement

**Table:**

Refinement on *F*^2^	Primary atom site location: structure-invariant direct methods
Least-squares matrix: full	Secondary atom site location: difference Fourier map
*R*[*F*^2^ > 2σ(*F*^2^)] = 0.046	Hydrogen site location: inferred from neighbouring sites
*wR*(*F*^2^) = 0.120	H atoms treated by a mixture of independent and constrained refinement
*S* = 1.06	*w* = 1/[σ^2^(*F*_o_^2^) + (0.0552*P*)^2^] where *P* = (*F*_o_^2^ + 2*F*_c_^2^)/3
6021 reflections	(Δ/σ)_max_ < 0.001
387 parameters	Δρ_max_ = 0.20 e Å^−^^3^
2 restraints	Δρ_min_ = −0.23 e Å^−^^3^

### Special details

**Table:**

Geometry. Bond distances, angles etc. have been calculated using the rounded fractional coordinates. All su's are estimated from the variances of the (full) variance-covariance matrix. The cell esds are taken into account in the estimation of distances, angles and torsion angles
Refinement. Refinement of F^2^ against ALL reflections. The weighted R-factor wR and goodness of fit S are based on F^2^, conventional R-factors R are based on F, with F set to zero for negative F^2^. The threshold expression of F^2^ > 2sigma(F^2^) is used only for calculating R-factors(gt) etc. and is not relevant to the choice of reflections for refinement. R-factors based on F^2^ are statistically about twice as large as those based on F, and R- factors based on ALL data will be even larger.

### Fractional atomic coordinates and isotropic or equivalent isotropic displacement parameters (Å^2^)

**Table:**

	*x*	*y*	*z*	*U*_iso_*/*U*_eq_	
O1	0.22974 (15)	−0.08495 (9)	−0.19945 (7)	0.0353 (4)	
N1	0.26603 (15)	−0.03201 (9)	−0.00509 (8)	0.0228 (4)	
N2	0.28634 (16)	−0.13393 (10)	−0.05950 (8)	0.0240 (4)	
N3	0.2868 (2)	−0.37222 (11)	−0.25733 (9)	0.0395 (5)	
C1	0.22947 (18)	0.03886 (11)	−0.04340 (9)	0.0214 (4)	
C2	0.2066 (2)	0.00985 (13)	−0.14333 (10)	0.0273 (5)	
C3	0.1577 (2)	0.09342 (13)	−0.17514 (10)	0.0322 (5)	
C4	0.1391 (2)	0.19509 (13)	−0.11610 (10)	0.0323 (5)	
C5	0.16559 (19)	0.22862 (12)	−0.01740 (10)	0.0258 (5)	
C6	0.1453 (2)	0.33609 (13)	0.04241 (11)	0.0323 (5)	
C7	0.1666 (2)	0.36735 (13)	0.13627 (11)	0.0343 (5)	
C8	0.2071 (2)	0.29038 (13)	0.17290 (10)	0.0318 (5)	
C9	0.2267 (2)	0.18361 (12)	0.11562 (10)	0.0265 (5)	
C10	0.20812 (18)	0.15051 (11)	0.01973 (9)	0.0221 (4)	
C11	0.31180 (18)	−0.21245 (11)	−0.02079 (9)	0.0221 (4)	
C12	0.31961 (19)	−0.32258 (12)	−0.08066 (9)	0.0246 (4)	
C13	0.3380 (2)	−0.40431 (12)	−0.04526 (10)	0.0307 (5)	
C14	0.3500 (2)	−0.37679 (13)	0.04877 (11)	0.0336 (5)	
C15	0.3436 (2)	−0.26761 (12)	0.10790 (10)	0.0302 (5)	
C16	0.32483 (19)	−0.18560 (12)	0.07411 (9)	0.0252 (4)	
C17	0.3020 (2)	−0.35134 (12)	−0.17923 (11)	0.0287 (5)	
O2	0.25056 (17)	−0.07164 (10)	0.29923 (7)	0.0412 (4)	
N4	0.23605 (16)	−0.01608 (10)	0.49355 (8)	0.0263 (4)	
N5	0.15976 (18)	−0.12048 (10)	0.43789 (8)	0.0287 (4)	
N6	−0.0106 (2)	−0.36198 (11)	0.23897 (9)	0.0409 (5)	
C18	0.3133 (2)	0.05720 (12)	0.45560 (10)	0.0259 (5)	
C19	0.3193 (2)	0.02819 (14)	0.35679 (10)	0.0320 (5)	
C20	0.4066 (2)	0.11518 (15)	0.32526 (11)	0.0381 (6)	
C21	0.4765 (2)	0.22096 (15)	0.38446 (11)	0.0391 (6)	
C22	0.4725 (2)	0.25414 (13)	0.48296 (11)	0.0317 (5)	
C23	0.5459 (2)	0.36558 (14)	0.54297 (13)	0.0426 (6)	
C24	0.5446 (2)	0.39650 (14)	0.63640 (13)	0.0428 (6)	
C25	0.4710 (2)	0.31589 (14)	0.67273 (11)	0.0391 (6)	
C26	0.3963 (2)	0.20554 (13)	0.61554 (10)	0.0319 (5)	
C27	0.3940 (2)	0.17243 (12)	0.51944 (10)	0.0272 (5)	
C28	0.0844 (2)	−0.19913 (12)	0.47635 (10)	0.0257 (5)	
C29	−0.0006 (2)	−0.30904 (12)	0.41620 (9)	0.0275 (5)	
C30	−0.0778 (2)	−0.39107 (13)	0.45099 (10)	0.0319 (5)	
C31	−0.0682 (2)	−0.36403 (13)	0.54521 (10)	0.0338 (5)	
C32	0.0176 (2)	−0.25528 (13)	0.60442 (10)	0.0328 (5)	
C33	0.0925 (2)	−0.17330 (13)	0.57078 (10)	0.0292 (5)	
C34	−0.0066 (2)	−0.33815 (12)	0.31768 (11)	0.0314 (5)	
H2	0.272 (2)	−0.1545 (14)	−0.1223 (6)	0.052 (5)*	
H3	0.13860	0.07610	−0.23830	0.0390*	
H4	0.10770	0.24650	−0.13970	0.0390*	
H6	0.11680	0.38740	0.01790	0.0390*	
H7	0.15390	0.43950	0.17520	0.0410*	
H8	0.22120	0.31110	0.23660	0.0380*	
H9	0.25270	0.13280	0.14120	0.0320*	
H13	0.34210	−0.47760	−0.08500	0.0370*	
H14	0.36250	−0.43140	0.07260	0.0400*	
H15	0.35200	−0.24960	0.17140	0.0360*	
H16	0.32090	−0.11260	0.11450	0.0300*	
H5	0.171 (3)	−0.1310 (16)	0.3780 (7)	0.075 (7)*	
H20	0.41490	0.09770	0.26230	0.0460*	
H21	0.52990	0.27530	0.36110	0.0470*	
H23	0.59660	0.41960	0.51880	0.0510*	
H24	0.59290	0.47120	0.67550	0.0510*	
H25	0.47210	0.33670	0.73640	0.0470*	
H26	0.34690	0.15250	0.64090	0.0380*	
H30	−0.13560	−0.46380	0.41090	0.0380*	
H31	−0.11910	−0.41850	0.56890	0.0410*	
H32	0.02470	−0.23750	0.66790	0.0390*	
H33	0.14840	−0.10060	0.61140	0.0350*	

### Atomic displacement parameters (Å^2^)

**Table:**

	*U*^11^	*U*^22^	*U*^33^	*U*^12^	*U*^13^	*U*^23^
O1	0.0424 (7)	0.0354 (7)	0.0234 (6)	0.0079 (5)	0.0014 (5)	0.0058 (5)
N1	0.0197 (6)	0.0200 (7)	0.0252 (7)	0.0027 (5)	0.0010 (5)	0.0052 (5)
N2	0.0251 (6)	0.0214 (7)	0.0227 (7)	0.0043 (5)	0.0006 (5)	0.0054 (6)
N3	0.0474 (9)	0.0325 (8)	0.0325 (8)	0.0039 (7)	0.0031 (6)	0.0074 (7)
C1	0.0166 (7)	0.0232 (8)	0.0238 (8)	0.0011 (6)	−0.0008 (6)	0.0095 (7)
C2	0.0234 (7)	0.0285 (9)	0.0265 (9)	0.0016 (6)	0.0009 (6)	0.0083 (7)
C3	0.0324 (8)	0.0399 (10)	0.0267 (9)	0.0072 (7)	−0.0018 (7)	0.0155 (8)
C4	0.0282 (8)	0.0364 (10)	0.0379 (10)	0.0053 (7)	−0.0016 (7)	0.0210 (8)
C5	0.0178 (7)	0.0281 (9)	0.0328 (9)	0.0029 (6)	−0.0002 (6)	0.0139 (7)
C6	0.0252 (8)	0.0265 (9)	0.0484 (11)	0.0055 (7)	0.0010 (7)	0.0177 (8)
C7	0.0278 (8)	0.0248 (9)	0.0456 (10)	0.0060 (7)	0.0062 (7)	0.0075 (8)
C8	0.0301 (8)	0.0303 (9)	0.0298 (9)	0.0030 (7)	0.0054 (7)	0.0069 (7)
C9	0.0261 (8)	0.0243 (8)	0.0291 (9)	0.0038 (6)	0.0027 (6)	0.0108 (7)
C10	0.0153 (7)	0.0219 (8)	0.0273 (8)	0.0008 (6)	0.0004 (6)	0.0088 (6)
C11	0.0181 (7)	0.0211 (8)	0.0259 (8)	0.0028 (6)	0.0017 (6)	0.0080 (6)
C12	0.0219 (7)	0.0220 (8)	0.0269 (8)	0.0028 (6)	0.0017 (6)	0.0065 (7)
C13	0.0300 (8)	0.0220 (8)	0.0363 (9)	0.0050 (7)	0.0018 (7)	0.0067 (7)
C14	0.0347 (9)	0.0284 (9)	0.0433 (10)	0.0074 (7)	0.0013 (7)	0.0195 (8)
C15	0.0311 (8)	0.0323 (9)	0.0282 (9)	0.0062 (7)	0.0013 (7)	0.0126 (7)
C16	0.0245 (7)	0.0232 (8)	0.0252 (8)	0.0044 (6)	0.0017 (6)	0.0062 (7)
C17	0.0284 (8)	0.0205 (8)	0.0320 (10)	0.0025 (6)	0.0037 (7)	0.0050 (7)
O2	0.0551 (8)	0.0418 (7)	0.0233 (6)	0.0147 (6)	0.0010 (5)	0.0060 (5)
N4	0.0251 (6)	0.0258 (7)	0.0259 (7)	0.0089 (6)	−0.0009 (5)	0.0057 (6)
N5	0.0338 (7)	0.0263 (8)	0.0235 (7)	0.0094 (6)	−0.0001 (6)	0.0051 (6)
N6	0.0539 (9)	0.0338 (8)	0.0313 (8)	0.0092 (7)	0.0007 (7)	0.0080 (7)
C18	0.0229 (7)	0.0304 (9)	0.0253 (8)	0.0099 (7)	0.0005 (6)	0.0094 (7)
C19	0.0297 (8)	0.0395 (10)	0.0279 (9)	0.0149 (7)	0.0015 (7)	0.0101 (8)
C20	0.0339 (9)	0.0548 (12)	0.0313 (9)	0.0139 (8)	0.0054 (7)	0.0205 (9)
C21	0.0290 (9)	0.0493 (11)	0.0477 (11)	0.0067 (8)	0.0043 (7)	0.0287 (9)
C22	0.0209 (7)	0.0348 (10)	0.0395 (10)	0.0049 (7)	−0.0011 (7)	0.0145 (8)
C23	0.0281 (9)	0.0403 (11)	0.0610 (13)	0.0001 (8)	−0.0067 (8)	0.0245 (10)
C24	0.0346 (9)	0.0293 (10)	0.0554 (12)	0.0027 (8)	−0.0123 (8)	0.0077 (9)
C25	0.0367 (9)	0.0383 (11)	0.0342 (10)	0.0112 (8)	−0.0100 (7)	0.0029 (8)
C26	0.0312 (8)	0.0320 (10)	0.0306 (9)	0.0096 (7)	−0.0025 (7)	0.0082 (8)
C27	0.0206 (7)	0.0299 (9)	0.0305 (9)	0.0093 (7)	−0.0009 (6)	0.0086 (7)
C28	0.0249 (8)	0.0266 (9)	0.0264 (8)	0.0109 (7)	−0.0003 (6)	0.0084 (7)
C29	0.0287 (8)	0.0285 (9)	0.0233 (8)	0.0117 (7)	−0.0031 (6)	0.0047 (7)
C30	0.0314 (8)	0.0253 (9)	0.0339 (9)	0.0063 (7)	−0.0050 (7)	0.0054 (7)
C31	0.0349 (9)	0.0308 (9)	0.0353 (10)	0.0040 (7)	−0.0028 (7)	0.0133 (8)
C32	0.0323 (9)	0.0379 (10)	0.0270 (9)	0.0068 (7)	−0.0018 (7)	0.0113 (8)
C33	0.0287 (8)	0.0259 (9)	0.0282 (9)	0.0066 (7)	−0.0031 (6)	0.0042 (7)
C34	0.0347 (9)	0.0244 (9)	0.0329 (10)	0.0096 (7)	−0.0023 (7)	0.0066 (7)

### Geometric parameters (Å, º)

**Table:**

O1—C2	1.261 (2)	C8—H8	0.9300
O2—C19	1.273 (2)	C9—H9	0.9300
N1—N2	1.3173 (17)	C13—H13	0.9300
N1—C1	1.3263 (19)	C14—H14	0.9300
N2—C11	1.397 (2)	C15—H15	0.9300
N3—C17	1.148 (2)	C16—H16	0.9300
N2—H2	0.916 (9)	C18—C27	1.459 (2)
N4—N5	1.3162 (18)	C18—C19	1.451 (2)
N4—C18	1.334 (2)	C19—C20	1.432 (3)
N5—C28	1.392 (2)	C20—C21	1.336 (3)
N6—C34	1.151 (2)	C21—C22	1.439 (2)
N5—H5	0.901 (12)	C22—C27	1.409 (2)
C1—C10	1.457 (2)	C22—C23	1.396 (3)
C1—C2	1.465 (2)	C23—C24	1.365 (3)
C2—C3	1.440 (2)	C24—C25	1.387 (3)
C3—C4	1.333 (2)	C25—C26	1.376 (2)
C4—C5	1.440 (2)	C26—C27	1.400 (2)
C5—C6	1.398 (2)	C28—C33	1.385 (2)
C5—C10	1.412 (2)	C28—C29	1.400 (2)
C6—C7	1.368 (2)	C29—C30	1.392 (2)
C7—C8	1.389 (2)	C29—C34	1.442 (2)
C8—C9	1.378 (2)	C30—C31	1.380 (2)
C9—C10	1.396 (2)	C31—C32	1.387 (2)
C11—C16	1.3903 (19)	C32—C33	1.376 (2)
C11—C12	1.401 (2)	C20—H20	0.9300
C12—C13	1.387 (2)	C21—H21	0.9300
C12—C17	1.443 (2)	C23—H23	0.9300
C13—C14	1.376 (2)	C24—H24	0.9300
C14—C15	1.386 (2)	C25—H25	0.9300
C15—C16	1.374 (2)	C26—H26	0.9300
C3—H3	0.9300	C30—H30	0.9300
C4—H4	0.9300	C31—H31	0.9300
C6—H6	0.9300	C32—H32	0.9300
C7—H7	0.9300	C33—H33	0.9300
			
N2—N1—C1	118.56 (12)	C14—C15—H15	119.00
N1—N2—C11	119.42 (11)	C16—C15—H15	120.00
C11—N2—H2	120.8 (11)	C11—C16—H16	120.00
N1—N2—H2	119.5 (11)	C15—C16—H16	120.00
N5—N4—C18	117.91 (12)	C19—C18—C27	119.88 (14)
N4—N5—C28	118.70 (12)	N4—C18—C27	116.30 (13)
N4—N5—H5	112.2 (13)	N4—C18—C19	123.82 (14)
C28—N5—H5	129.0 (13)	O2—C19—C18	121.45 (16)
N1—C1—C10	116.45 (12)	O2—C19—C20	120.47 (14)
C2—C1—C10	119.75 (13)	C18—C19—C20	118.08 (15)
N1—C1—C2	123.79 (13)	C19—C20—C21	121.38 (15)
O1—C2—C1	121.04 (14)	C20—C21—C22	122.62 (17)
O1—C2—C3	121.26 (13)	C21—C22—C27	119.33 (15)
C1—C2—C3	117.70 (14)	C23—C22—C27	119.46 (15)
C2—C3—C4	121.48 (14)	C21—C22—C23	121.21 (16)
C3—C4—C5	122.80 (15)	C22—C23—C24	121.13 (17)
C6—C5—C10	119.37 (13)	C23—C24—C25	119.63 (17)
C4—C5—C6	121.21 (15)	C24—C25—C26	120.72 (15)
C4—C5—C10	119.40 (14)	C25—C26—C27	120.53 (16)
C5—C6—C7	121.32 (16)	C22—C27—C26	118.51 (15)
C6—C7—C8	119.39 (15)	C18—C27—C26	122.81 (15)
C7—C8—C9	120.57 (14)	C18—C27—C22	118.67 (13)
C8—C9—C10	120.99 (14)	N5—C28—C29	117.98 (13)
C1—C10—C9	122.83 (13)	N5—C28—C33	122.77 (14)
C5—C10—C9	118.36 (13)	C29—C28—C33	119.25 (15)
C1—C10—C5	118.81 (12)	C28—C29—C30	120.34 (13)
N2—C11—C12	118.15 (12)	C30—C29—C34	119.64 (14)
N2—C11—C16	122.33 (13)	C28—C29—C34	120.02 (14)
C12—C11—C16	119.50 (14)	C29—C30—C31	119.69 (15)
C11—C12—C17	119.62 (14)	C30—C31—C32	119.71 (16)
C13—C12—C17	120.29 (14)	C31—C32—C33	121.05 (14)
C11—C12—C13	120.06 (12)	C28—C33—C32	119.96 (15)
C12—C13—C14	119.88 (14)	N6—C34—C29	179.50 (17)
C13—C14—C15	119.98 (16)	C19—C20—H20	119.00
C14—C15—C16	120.98 (14)	C21—C20—H20	119.00
C11—C16—C15	119.60 (14)	C20—C21—H21	119.00
N3—C17—C12	178.63 (17)	C22—C21—H21	119.00
C4—C3—H3	119.00	C22—C23—H23	119.00
C2—C3—H3	119.00	C24—C23—H23	119.00
C5—C4—H4	119.00	C23—C24—H24	120.00
C3—C4—H4	119.00	C25—C24—H24	120.00
C5—C6—H6	119.00	C24—C25—H25	120.00
C7—C6—H6	119.00	C26—C25—H25	120.00
C6—C7—H7	120.00	C25—C26—H26	120.00
C8—C7—H7	120.00	C27—C26—H26	120.00
C9—C8—H8	120.00	C29—C30—H30	120.00
C7—C8—H8	120.00	C31—C30—H30	120.00
C10—C9—H9	119.00	C30—C31—H31	120.00
C8—C9—H9	120.00	C32—C31—H31	120.00
C12—C13—H13	120.00	C31—C32—H32	119.00
C14—C13—H13	120.00	C33—C32—H32	119.00
C13—C14—H14	120.00	C28—C33—H33	120.00
C15—C14—H14	120.00	C32—C33—H33	120.00
			
C1—N1—N2—C11	−175.64 (12)	C11—C12—C13—C14	−0.5 (2)
N2—N1—C1—C2	1.2 (2)	C17—C12—C13—C14	−178.41 (14)
N2—N1—C1—C10	179.96 (12)	C12—C13—C14—C15	0.0 (2)
N1—N2—C11—C12	175.62 (12)	C13—C14—C15—C16	0.2 (2)
N1—N2—C11—C16	−2.8 (2)	C14—C15—C16—C11	0.1 (2)
N5—N4—C18—C27	179.18 (13)	N4—C18—C19—O2	−0.5 (2)
C18—N4—N5—C28	177.81 (13)	N4—C18—C19—C20	180.00 (15)
N5—N4—C18—C19	−0.5 (2)	C27—C18—C19—O2	179.83 (14)
N4—N5—C28—C29	178.40 (13)	C27—C18—C19—C20	0.3 (2)
N4—N5—C28—C33	−2.4 (2)	N4—C18—C27—C22	−178.20 (13)
C10—C1—C2—O1	177.94 (13)	N4—C18—C27—C26	1.6 (2)
C10—C1—C2—C3	−1.8 (2)	C19—C18—C27—C22	1.5 (2)
N1—C1—C2—O1	−3.3 (2)	C19—C18—C27—C26	−178.72 (14)
N1—C1—C2—C3	176.96 (13)	O2—C19—C20—C21	178.79 (15)
C2—C1—C10—C9	179.57 (13)	C18—C19—C20—C21	−1.7 (2)
N1—C1—C10—C5	−178.85 (12)	C19—C20—C21—C22	1.2 (2)
N1—C1—C10—C9	0.7 (2)	C20—C21—C22—C23	−179.67 (15)
C2—C1—C10—C5	0.0 (2)	C20—C21—C22—C27	0.7 (2)
O1—C2—C3—C4	−177.83 (15)	C21—C22—C23—C24	−179.01 (14)
C1—C2—C3—C4	1.9 (2)	C27—C22—C23—C24	0.6 (2)
C2—C3—C4—C5	−0.2 (2)	C21—C22—C27—C18	−2.0 (2)
C3—C4—C5—C10	−1.8 (2)	C21—C22—C27—C26	178.21 (14)
C3—C4—C5—C6	179.86 (15)	C23—C22—C27—C18	178.35 (14)
C6—C5—C10—C9	0.6 (2)	C23—C22—C27—C26	−1.4 (2)
C4—C5—C10—C1	1.8 (2)	C22—C23—C24—C25	0.7 (2)
C4—C5—C6—C7	178.66 (14)	C23—C24—C25—C26	−1.1 (2)
C10—C5—C6—C7	0.3 (2)	C24—C25—C26—C27	0.3 (2)
C4—C5—C10—C9	−177.84 (13)	C25—C26—C27—C18	−178.79 (14)
C6—C5—C10—C1	−179.82 (13)	C25—C26—C27—C22	1.0 (2)
C5—C6—C7—C8	−0.7 (2)	N5—C28—C29—C30	179.85 (13)
C6—C7—C8—C9	0.3 (2)	N5—C28—C29—C34	0.6 (2)
C7—C8—C9—C10	0.6 (2)	C33—C28—C29—C30	0.7 (2)
C8—C9—C10—C1	179.42 (14)	C33—C28—C29—C34	−178.65 (14)
C8—C9—C10—C5	−1.0 (2)	N5—C28—C33—C32	−179.13 (14)
C16—C11—C12—C13	0.7 (2)	C29—C28—C33—C32	0.0 (2)
C16—C11—C12—C17	178.68 (13)	C28—C29—C30—C31	−0.8 (2)
N2—C11—C12—C17	0.2 (2)	C34—C29—C30—C31	178.51 (14)
N2—C11—C12—C13	−177.75 (13)	C29—C30—C31—C32	0.2 (2)
C12—C11—C16—C15	−0.5 (2)	C30—C31—C32—C33	0.4 (2)
N2—C11—C16—C15	177.89 (13)	C31—C32—C33—C28	−0.6 (2)

### Hydrogen-bond geometry (Å, º)

**Table:**

*D*—H···*A*	*D*—H	H···*A*	*D*···*A*	*D*—H···*A*
N2—H2···O1	0.92 (1)	1.80 (2)	2.5380 (16)	136 (2)
N5—H5···O2	0.90 (1)	1.72 (2)	2.5277 (17)	147 (2)
C21—H21···N3^i^	0.93	2.61	3.509 (2)	162
C30—H30···N3^ii^	0.93	2.60	3.487 (2)	159
C32—H32···O1^iii^	0.93	2.49	3.1994 (18)	133

Symmetry codes: (i) −*x*+1, −*y*, −*z*; (ii) −*x*, −*y*−1, −*z*; (iii) *x*, *y*, *z*+1.
